# County-level social, preventive care, and behavioral correlates of colorectal cancer mortality in the United States: a nationwide ecological analysis

**DOI:** 10.3389/fonc.2026.1844633

**Published:** 2026-07-28

**Authors:** Kaide Xia, Li Luo, Chengdu Fan, Hexuan Zhang, Xiuju Yang, Chaohuang Lin

**Affiliations:** 1 Laboratory of Molecular Genetic Diagnostics, Guiyang Maternal and Child Health Care Hospital, Guiyang Children’s Hospital, Guiyang, China; 2 Department of Clinical Laboratory, The Second People’s Hospital of Guiyang, Guiyang, China; 3 Department of Anesthesiology, The Affiliated Cancer Hospital of Guizhou Medical University, Guiyang, China; 4 Department of Gastrointestinal Surgery, The Affiliated Cancer Hospital of Guizhou Medical University, Guiyang, China

**Keywords:** broadband access, cancer mortality, colorectal cancer, geographic disparities, social determinants of health

## Abstract

**Background:**

Geographic disparities in colorectal cancer (CRC) outcomes remain substantial across U.S. counties. Associations of social conditions, preventive care, behavioral risk environments, and rurality with CRC mortality remain incompletely characterized in analyses addressing state-level clustering and spatial dependence.

**Methods:**

We linked CDC PLACES, American Community Survey, United States Cancer Statistics, and 2023 National Center for Health Statistics rurality data in a nationwide county-level ecological study. Outcomes were age-adjusted CRC mortality and all-cancer mortality during 2019–2023 and CRC incidence during 2018–2022. Population-weighted core-adjusted linear models evaluated each exposure separately, adjusting for the county proportion aged ≥65 years, nonmetropolitan status where applicable, and state fixed effects. Standard errors were clustered by state using CR2 estimation with Satterthwaite degrees of freedom.

**Results:**

The ACS-complete dataset included 3,142 counties; 3,134 remained after linkage to the 2023 NCHS rurality classification. Final fixed samples included 2,126 counties for CRC mortality, 3,005 for all-cancer mortality, and 2,628 for CRC incidence. In core-adjusted CRC mortality models, greater broadband deprivation (β = 1.82, 95% CI 1.44 to 2.20), higher current smoking prevalence (β =2.08, 95% CI 1.74 to 2.42), and nonmetropolitan status (β=2.73, 95% CI 2.35 to 3.11) were associated with higher mortality. Higher CRC screening uptake (β = −1.64, 95% CI −1.97 to −1.31) and recent dental visit prevalence (β = −1.84, 95% CI −2.27 to −1.40) were associated with lower mortality. Key associations were directionally consistent in log-rate and matched spatial error model sensitivity analyses, with similar patterns for all-cancer mortality and CRC incidence.

**Conclusions:**

CRC mortality was associated with adverse social and behavioral profiles, lower preventive care engagement, and nonmetropolitan residence. Overlap across CRC mortality, all-cancer mortality, and CRC incidence suggests that these indicators may reflect broader geographic cancer disadvantage rather than CRC-specific mechanisms. Integrated structural, preventive care, behavioral, and rural health strategies may help reduce county-level disparities in CRC outcomes.

## 1 Introduction

Colorectal cancer (CRC) remains a major contributor to cancer burden in the United States, yet substantial geographic disparities in CRC outcomes persist across counties (1, 2). Although population-level declines in CRC incidence and mortality have been observed over time, these improvements have not been evenly distributed, and some counties continue to experience disproportionately high CRC burden (3, 4). Understanding the contextual factors associated with these county-level disparities is important for identifying high-burden communities and informing geographically targeted cancer control strategies (5, 6).

County-level disparities in CRC outcomes likely reflect the combined influence of structural disadvantage, access to preventive care, local health behavior environments, and rurality (7). Traditional social determinants of health, including poverty, unemployment, and lower educational attainment, have been associated with lower cancer screening uptake and reduced access to timely care (8, 9). At the same time, preventive and follow-up care increasingly depends on digital infrastructure, including patient portals, telehealth communication, appointment coordination, access to health information, and navigation of multistep screening pathways (10). Broadband access may therefore serve as a contextual marker of healthcare connectivity and care navigation capacity in contemporary CRC prevention and management (11). However, its county-level association with CRC outcomes, together with correlated socioeconomic, preventive care, behavioral, and rurality indicators, remains incompletely characterized (12).

It is also important to determine whether county-level correlates of CRC mortality overlap with broader cancer mortality and CRC incidence patterns. Comparing CRC mortality with all-cancer mortality can help characterize whether observed associations reflect broader geographic cancer disadvantage, whereas examining CRC incidence can indicate whether similar contextual patterns extend across the CRC disease continuum (13–17). These comparisons should be interpreted as descriptive ecological contrasts rather than tests of disease-specific causal mechanisms.

In this nationwide county-level ecological study, we linked county-level data from CDC PLACES, the American Community Survey, United States Cancer Statistics, and the 2023 National Center for Health Statistics urban-rural classification scheme. Our objectives were to: (1) characterize county-level variation in CRC mortality and describe differences between high- and low-burden counties; (2) estimate core-adjusted associations of social, preventive care, behavioral, and rurality indicators with CRC mortality; and (3) compare key associations across CRC mortality, all-cancer mortality, and CRC incidence. We hypothesized that adverse social and behavioral profiles, lower preventive care engagement, and nonmetropolitan county status would be associated with higher county-level CRC burden.

## 2 Methods

### 2.1 Study design and data sources

We conducted a nationwide county-level ecological study of U.S. counties using linked public datasets. The county-level analytic base was constructed from the 2023 CDC PLACES county GIS-friendly release and the American Community Survey (ACS) 2017–2021 county-level non-medical factor measures dataset (18, 19). During preprocessing, variable names were standardized, county identifiers were harmonized, and ACS measures were reshaped from long to wide format before linkage with PLACES data by county Federal Information Processing Standards code. Counties with complete ACS-derived measures were retained for the initial merged dataset.

Rurality was classified using the 2023 National Center for Health Statistics Urban-Rural Classification Scheme for Counties. Counties classified in categories 1–4 were considered metropolitan, whereas counties classified in categories 5–6 were considered nonmetropolitan. Eight counties with legacy Connecticut county FIPS codes did not have direct matches in the 2023 NCHS classification file and were excluded before construction of outcome-specific analytic samples (20).

Cancer outcomes were derived from county-level United States Cancer Statistics ASCII files. Records were restricted to county-level observations for all racial/ethnic groups and both sexes. For all-cancer mortality, records were restricted to overall cancer mortality across all sites. For CRC outcomes, records were restricted to malignancies of the colon and rectum and subsequently separated into incidence and mortality datasets. Suppressed or unavailable age-adjusted rates were treated as missing. County FIPS codes were extracted from the USCS area field before linkage with the PLACES/ACS/NCHS analytic dataset (21).

This study used publicly available, de-identified county-level data and did not involve direct participant contact. Therefore, institutional review board approval and informed consent were not required.

### 2.2 Outcomes

The primary outcome was county-level age-adjusted CRC mortality for 2019–2023 combined. The secondary outcome was county-level age-adjusted all-cancer mortality for 2019–2023 combined. The exploratory outcome was county-level age-adjusted CRC incidence for 2018–2022 combined. Outcome-specific analytic datasets were created after merging these USCS outcomes with the county-level PLACES/ACS/NCHS analytic base. The outcome windows differed because publicly available county-level USCS files were released for different periods. CRC incidence was assessed during 2018–2022, whereas mortality outcomes were assessed during 2019–2023. Each outcome was analyzed separately in an outcome-specific analytic sample, and cross-outcome comparisons were interpreted as descriptive ecological contrasts rather than formal tests of disease-specific differences.

### 2.3 County-level correlates

County-level explanatory variables were grouped *a priori* into three domains (13, 22). The social determinants domain included poverty below 150% of the federal poverty level, no high school diploma, unemployment, high housing cost burden, crowded housing, single-parent households, racial/ethnic minority composition, and lack of broadband internet access. The healthcare access and preventive care domain included lack of health insurance, routine checkup, recent dental visit, and up-to-date CRC screening. The behavioral risk indicator domain included obesity, current smoking, binge drinking, short sleep duration, and physical inactivity. Continuous exposure variables were standardized to z scores. This standardization facilitated comparison of association magnitudes across county-level indicators with different distributions and variability, such that regression coefficients represented the absolute difference in the county-level outcome associated with a 1-standard-deviation higher prevalence of the corresponding exposure. The proportion of county residents aged ≥65 years was included as a demographic covariate. Rurality was represented by nonmetropolitan versus metropolitan county status.

### 2.4 Study sample and descriptive analyses

The ACS-complete merged dataset initially included 3,142 counties. After exclusion of eight counties without direct matches in the 2023 NCHS rurality classification, the county analytic base included 3,134 counties. Outcome-specific samples were then defined according to availability of each cancer outcome. CRC mortality was available for 2,188 counties, all-cancer mortality for 3,072 counties, and CRC incidence for 2,694 counties.

**Figure 1:**
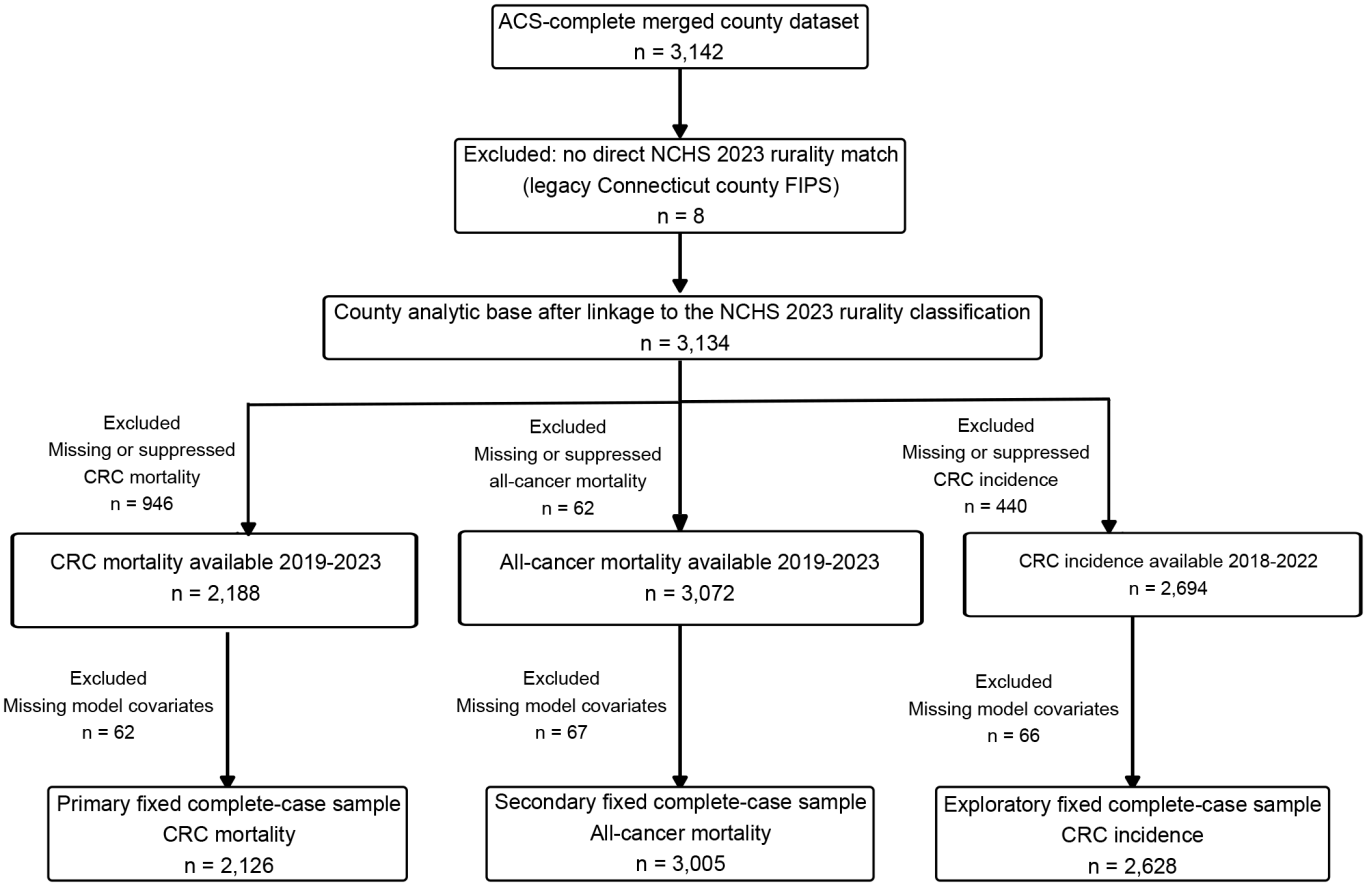
Study flow diagram. The ACS-complete merged county dataset included 3,142 counties. Eight counties with legacy Connecticut county FIPS codes lacked direct matches in the 2023 National Center for Health Statistics rurality classification and were excluded, leaving 3,134 counties in the county analytic base. Outcome-specific exclusions reflected unavailable or suppressed cancer outcome data and missing variables required for the fixed analytic samples. Final samples included 2,126 counties for CRC mortality, 3,005 counties for all-cancer mortality, and 2,628 counties for CRC incidence.

To ensure comparability of estimates across individual-exposure models within each outcome, we constructed outcome-specific fixed complete-case analytic samples using all variables required by the planned analytic framework. Within each outcome, the same fixed complete-case sample, defined by complete data on all candidate exposure variables and core covariates, was used for every individual-exposure model. The final fixed analytic samples included 2,126 counties for CRC mortality, 3,005 counties for all-cancer mortality, and 2,628 counties for CRC incidence. The study flow and outcome-specific exclusions are shown in **Figure 1**.

County-level characteristics were summarized across quartiles of CRC mortality burden in the final primary analytic sample. Geographic distributions of CRC mortality, all-cancer mortality, and CRC incidence were visualized using county-level maps.

### 2.5 Statistical analysis

The primary analyses used population-weighted linear regression models with state fixed effects. Because the outcomes were county-level age-adjusted rates rather than individual-level event counts, coefficients were interpreted as absolute differences in county-level rates per 100,000 population. County population was used as the analytic weight so that associations were estimated with greater influence from counties contributing larger population denominators. State fixed effects were included to account for time-invariant state-level differences in cancer control policies, healthcare systems, data-reporting context, and other unmeasured state characteristics that could confound cross-county associations. Population-weighted linear regression was selected because the publicly available outcomes were county-level age-adjusted rates rather than individual-level data or raw event counts analyzed with population offsets; this approach provides directly interpretable absolute differences in rates per 100,000 population. Poisson and negative binomial models were therefore not used as primary models. State fixed effects were preferred to random-effects multilevel models because the aim was to estimate within-state county-level associations while controlling for unmeasured, time-invariant state context without assuming that state effects were independent of county-level exposures. Geographically weighted regression was not used as the primary approach because the objective was to estimate national average associations rather than location-specific coefficients. Residual spatial dependence was evaluated using Moran’s I and matched spatial error model sensitivity analyses.

For each outcome, each focal exposure was evaluated in a separate core-adjusted individual-exposure model. Continuous exposure variables were standardized to 1-standard-deviation increments. Primary models adjusted for the county proportion aged ≥65 years, nonmetropolitan county status, and state fixed effects. For the rurality model, nonmetropolitan county status was the focal exposure; therefore, the model adjusted for age and state fixed effects but did not self-adjust for rurality.

Standard errors were clustered at the state level using the CR2 variance estimator, and inference was based on Satterthwaite degrees of freedom (23, 24). Multicollinearity was assessed using variance inflation factors for focal exposure terms in the core-adjusted models. Supplementary sequential conditional-association models were estimated for CRC mortality to transparently describe changes in estimates across nested domain-block specifications. Model 1 included social determinants, the county proportion aged ≥65 years, nonmetropolitan county status, and state fixed effects. Model 2 additionally included healthcare access and preventive care indicators. Model 3 additionally included behavioral risk indicators. Because social, preventive care, and behavioral indicators were correlated at the county level, these sequential models were treated as supplementary conditional-association analyses and were not used as the primary basis for variable-level inference. No formal mediation analysis was undertaken, and changes in estimates across the sequential models were not interpreted as evidence of mediation, confounding control, or independent explanatory power.

Sensitivity analyses used the same core-adjusted individual-exposure specifications with log(rate + 0.1) as the outcome, with estimates expressed as percentage changes in county-level rates. Residual spatial autocorrelation was assessed using Moran’s I for the five prespecified principal exposure models. For five prespecified principal exposures—lack of broadband internet access, up-to-date CRC screening, recent dental visits, current smoking, and nonmetropolitan county status—we fitted matched spatial error models using queen-contiguity county neighbor structures. These models used the same outcome, focal exposure, covariates, state fixed effects, and population weights as the corresponding primary models (25).

All analyses were performed in R. Two-sided P values <0.05 were considered statistically significant.

## 3 Results

### 3.1 Study sample, county characteristics, and geographic distribution

The ACS-complete merged dataset included 3,142 counties. Eight counties with legacy Connecticut county FIPS codes did not have direct matches in the 2023 National Center for Health Statistics rurality classification and were excluded, leaving a county analytic base of 3,134 counties. CRC mortality data were available for 2,188 counties, all-cancer mortality data for 3,072 counties, and CRC incidence data for 2,694 counties. Counties with unavailable or suppressed outcome data accounted for 946 exclusions from the CRC mortality analysis, 62 exclusions from the all-cancer mortality analysis, and 440 exclusions from the CRC incidence analysis. After additional exclusions for missing variables required for the outcome-specific fixed analytic samples, the final samples included 2,126 counties for CRC mortality, 3,005 counties for all-cancer mortality, and 2,628 counties for CRC incidence (**Figure 1**).

County-level characteristics across quartiles of CRC mortality burden are summarized in **Table 1**. Compared with counties in the lowest CRC mortality quartile, counties in the highest quartile had a less favorable social, preventive care, behavioral, and rurality profile. Specifically, counties in the highest quartile had a higher prevalence of poverty below 150% of the federal poverty level (29.31% vs 18.69%), lack of broadband internet access (23.35% vs 13.28%), and current smoking (21.98% vs 15.44%), whereas CRC screening uptake was lower (66.56% vs 70.18%; all P <0.001). Recent dental visit prevalence was also lower in the highest than in the lowest mortality quartile (53.82% vs 65.31%; P <0.001).

**Table 1:** County-level characteristics by quartiles of CRC mortality in the final primary analytic sample.

Characteristic	Overall	Q1 (Lowest)	Q2	Q3	Q4 (Highest)	P value
CRC Mortality Rate (per 100,000)	15.56 (4.45)	10.93 (1.14)	13.64 (0.63)	16.12 (0.86)	21.58 (3.90)	<0.001
Age ≥65 Years (%)	18.31 (4.10)	17.57 (4.49)	18.24 (4.48)	18.64 (3.80)	18.78 (3.45)	<0.001
Crowded Housing (%)	2.28 (1.78)	2.36 (1.93)	2.24 (1.55)	2.19 (1.62)	2.35 (1.98)	0.430
Single-Parent Households (%)	6.11 (2.02)	5.51 (1.56)	6.14 (1.86)	6.24 (2.04)	6.55 (2.41)	<0.001
Lack of Broadband Internet Access (%)	18.17 (7.20)	13.28 (5.52)	16.51 (5.51)	19.56 (6.68)	23.35 (6.85)	<0.001
High Housing Cost Burden (%)	23.11 (4.59)	24.16 (4.72)	23.78 (4.65)	22.59 (4.32)	21.92 (4.33)	<0.001
Racial/Ethnic Minority Population (%)	25.20 (19.49)	25.60 (18.27)	25.39 (18.83)	23.59 (18.88)	26.21 (21.76)	0.019
Income Below 150% of Poverty (%)	23.91 (7.96)	18.69 (6.74)	22.60 (6.28)	25.07 (6.85)	29.31 (7.92)	<0.001
No High School Diploma (%)	11.93 (5.38)	9.01 (4.77)	10.93 (4.56)	12.61 (4.78)	15.17 (5.40)	<0.001
Unemployment (%)	5.40 (2.11)	4.84 (1.62)	5.25 (1.70)	5.50 (2.05)	6.02 (2.71)	<0.001
Lack of Health Insurance (%)	11.83 (5.18)	9.57 (4.00)	11.46 (4.41)	12.32 (5.21)	13.99 (5.88)	<0.001
Routine Checkup (%)	73.03 (4.35)	71.63 (5.07)	72.73 (4.52)	73.79 (3.72)	73.99 (3.53)	<0.001
Up-to-Date Colorectal Cancer Screening (%)	68.44 (4.77)	70.18 (5.38)	68.86 (4.74)	68.15 (4.13)	66.56 (4.00)	<0.001
Recent Dental Visit (%)	59.50 (7.32)	65.31 (5.83)	60.96 (5.72)	57.92 (5.81)	53.82 (6.60)	<0.001
Obesity (%)	37.26 (4.56)	33.55 (4.79)	36.87 (3.77)	38.53 (3.21)	40.11 (3.47)	<0.001
Current Smoking (%)	18.84 (4.02)	15.44 (3.39)	18.08 (2.98)	19.88 (2.93)	21.98 (3.54)	<0.001
Binge Drinking (%)	17.25 (2.49)	18.22 (2.64)	17.54 (2.29)	17.02 (2.27)	16.23 (2.30)	<0.001
Short Sleep Duration (%)	34.83 (3.51)	32.53 (3.14)	34.54 (3.17)	35.65 (3.13)	36.59 (3.22)	<0.001
Physical Inactivity (%)	26.48 (5.18)	22.21 (4.30)	25.45 (3.99)	27.81 (3.97)	30.46 (4.52)	<0.001
Nonmetropolitan Counties, n (%)	1124 (52.9%)	153 (28.8%)	237 (44.5%)	315 (59.3%)	419 (78.9%)	<0.001

Values are mean (SD) unless otherwise indicated. P values were calculated using Kruskal-Wallis tests for continuous variables and chi-square tests for nonmetropolitan county status. Quartiles were defined using the CRC mortality rate in the final primary fixed complete-case sample (n =2,126).

**Figure 2:**
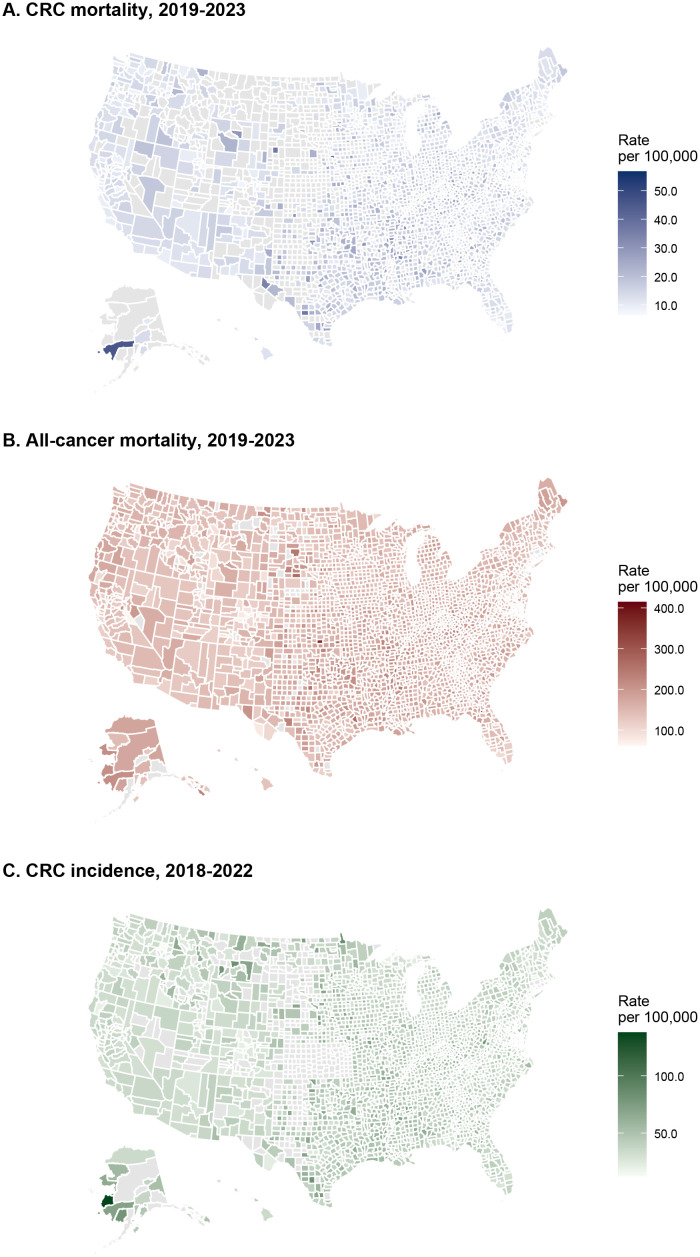
Geographic distribution of county-level cancer outcomes in the United States. **(A)** shows age-adjusted colorectal cancer mortality rates for 2019–2023; **(B)** shows age-adjusted all-cancer mortality rates for 2019–2023; and **(C)** shows age-adjusted colorectal cancer incidence rates for 2018–2022. Rates are shown per 100,000 population using outcome-specific scales. Grey counties had unavailable or suppressed outcome data.

Geographic patterns of county-level cancer outcomes are shown in **Figure 2**. CRC mortality, all-cancer mortality, and CRC incidence each displayed substantial geographic heterogeneity. The patterns of CRC mortality and all-cancer mortality partially overlapped, whereas CRC incidence did not completely mirror the distribution of CRC mortality. Because the maps use outcome-specific rate scales, the panels describe within-outcome geographic variation rather than directly comparable absolute color intensities across outcomes. Counties with unavailable or suppressed outcome data are shown in grey.

### 3.2 Core-adjusted associations with CRC mortality

**Figure 3:**
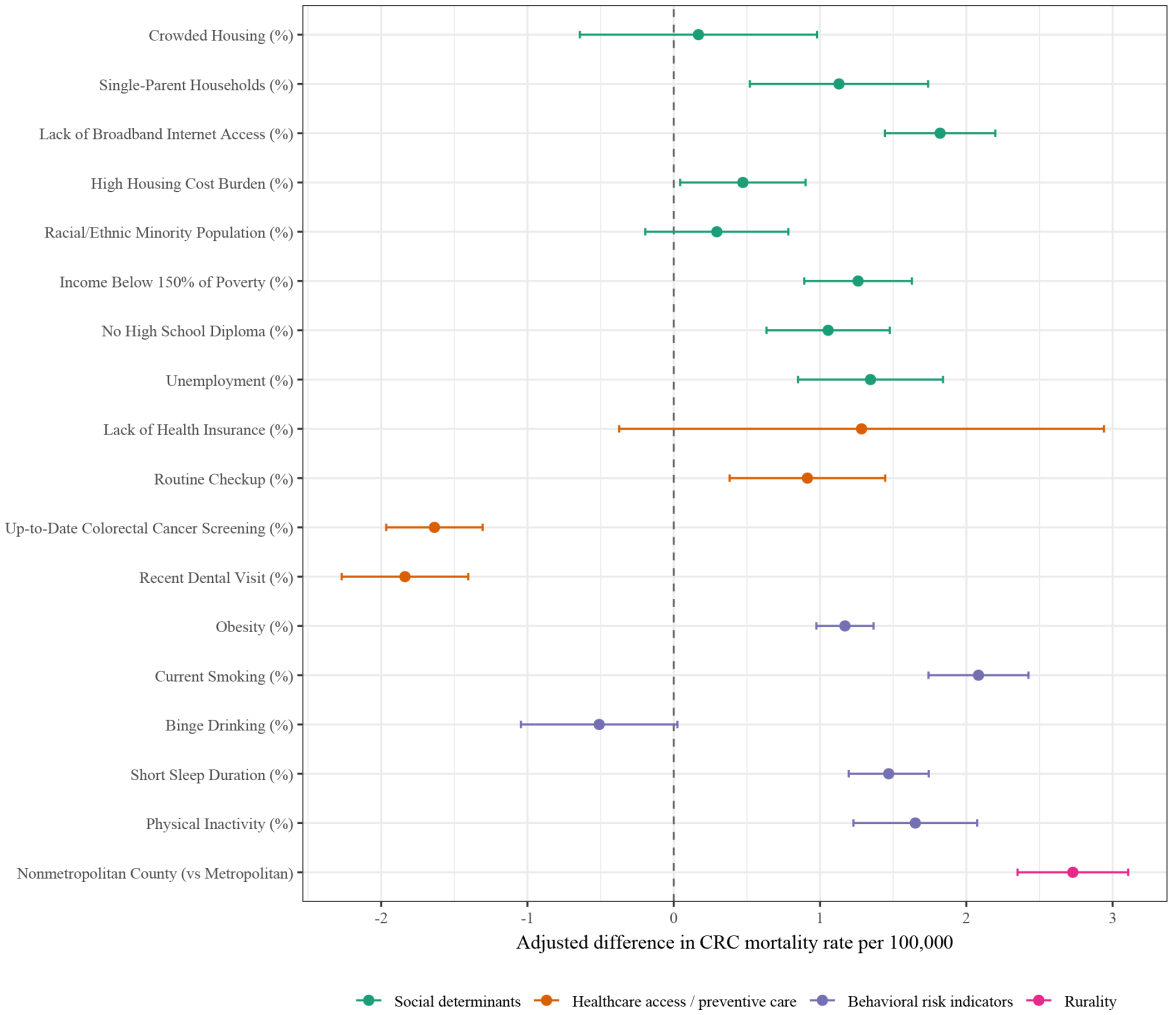
Core-adjusted associations with county-level colorectal cancer mortality. Each exposure was estimated in a separate population-weighted core-adjusted linear regression model with state fixed effects and state-clustered CR2 standard errors. Continuous exposures are expressed per 1-SD higher county-level prevalence. Nonmetropolitan county status compares nonmetropolitan with metropolitan counties. Models adjusted for the county proportion aged ≥65 years and nonmetropolitan county status where applicable; the rurality model adjusted for age and state fixed effects but did not self-adjust for rurality. Points indicate β coefficients and horizontal bars indicate 95% confidence intervals. β coefficients represent absolute differences in county-level colorectal cancer mortality rates per 100,000 population.

Core-adjusted associations between county-level factors and CRC mortality are presented in **Table 2**; **Figure 3**. Each exposure was estimated in a separate population-weighted model adjusted for the county proportion aged ≥65 years, nonmetropolitan county status where applicable, and state fixed effects.

**Table 2:** Core-adjusted associations of county-level factors with CRC mortality (n = 2,126 counties across 49 state clusters).

Domain	County-level correlate†	β (95% CI), per 100,000	P value
Social determinants			
	Crowded housing	0.17 (−0.64 to 0.98)	0.447
	Single-parent households	1.13 (0.52 to 1.74)	0.005
	Lack of broadband internet access	1.82 (1.44 to 2.20)	<0.001
	High housing cost burden	0.47 (0.04 to 0.90)	0.038
	Racial/ethnic minority population	0.29 (−0.19 to 0.78)	0.198
	Income below 150% of the federal poverty level	1.26 (0.89 to 1.63)	<0.001
	No high school diploma	1.06 (0.63 to 1.48)	0.005
	Unemployment	1.34 (0.85 to 1.84)	<0.001
Healthcare access and preventive care			
	Lack of health insurance	1.28 (−0.37 to 2.94)	0.082
	Routine checkup‡	0.91 (0.38 to 1.45)	0.009
	Up-to-date colorectal cancer screening	−1.64 (−1.97 to −1.31)	<0.001
	Recent dental visit	−1.84 (−2.27 to −1.40)	<0.001
Behavioral risk indicators			
	Obesity	1.17 (0.97 to 1.37)	<0.001
	Current smoking	2.08 (1.74 to 2.42)	<0.001
	Binge drinking	−0.51 (−1.04 to 0.03)	0.058
	Short sleep duration	1.47 (1.20 to 1.74)	<0.001
	Physical inactivity	1.65 (1.23 to 2.07)	<0.001
Rurality			
	Nonmetropolitan county versus metropolitan county	2.73 (2.35 to 3.11)	<0.001

CI, confidence interval; CRC, colorectal cancer.

Each row was estimated in a separate population-weighted core-adjusted linear regression model. Models adjusted for the county proportion aged ≥65 years, nonmetropolitan county status, and state fixed effects. The rurality model adjusted for age and state fixed effects but did not self-adjust for rurality. Standard errors were clustered at the state level using the CR2 variance estimator, and inference was based on Satterthwaite degrees of freedom.

^†^ Continuous county-level correlates were standardized to 1-SD increments. β coefficients represent absolute differences in age-adjusted county-level CRC mortality rates per 100,000 population associated with a 1-SD higher prevalence of the corresponding correlate. For rurality, the estimate compares nonmetropolitan with metropolitan counties. ‡ Routine checkup showed high focal-term variance inflation factors across outcomes and should be interpreted cautiously; see **Supplementary Table 3**.

Several social determinants were associated with higher CRC mortality. A 1-SD higher prevalence of lack of broadband internet access was associated with a 1.82-per-100,000 higher CRC mortality rate (95% CI 1.44 to 2.20; P <0.001). Higher prevalence of single-parent households, poverty below 150% of the federal poverty level, no high school diploma, unemployment, and high housing cost burden was also associated with higher CRC mortality.

Within the healthcare access and preventive care domain, higher up-to-date CRC screening prevalence was associated with lower CRC mortality (β = −1.64, 95% CI −1.97 to −1.31; P <0.001), as was higher recent dental visit prevalence (β = −1.84, 95% CI −2.27 to −1.40; P <0.001). Higher current smoking prevalence was associated with higher CRC mortality (β = 2.08, 95% CI 1.74 to 2.42; P <0.001). Obesity, short sleep duration, and physical inactivity were also positively associated with CRC mortality. Nonmetropolitan counties had higher CRC mortality rates than metropolitan counties (β = 2.73, 95% CI 2.35 to 3.11; P <0.001).

Routine checkup prevalence was positively associated with CRC mortality; however, the focal exposure term showed high VIF values across outcomes, and this estimate should therefore be interpreted cautiously. **Figure 3** displays the full pattern of exposure-wise core-adjusted associations.

### 3.3 Core-adjusted associations with all-cancer mortality

Core-adjusted associations with all-cancer mortality are presented in **Table 3**. Several key associations were directionally consistent with the CRC mortality analysis. Higher lack of broadband internet access was associated with higher all-cancer mortality (β = 12.28, 95% CI 7.98 to 16.57; P <0.001). Higher current smoking prevalence was also strongly associated with higher all-cancer mortality (β = 19.95, 95% CI 18.08 to 21.82; P <0.001).

**Table 3:** Core-adjusted associations of county-level factors with all-cancer mortality (n = 3,005 counties across 49 state clusters).

Domain	County-level correlate†	β (95% CI), per 100,000	P value
Social determinants			
	Crowded housing	−3.66 (−9.58 to 2.26)	0.115
	Single-parent households	8.95 (4.25 to 13.65)	0.004
	Lack of broadband internet access	12.28 (7.98 to 16.57)	<0.001
	High housing cost burden	2.94 (−1.09 to 6.98)	0.111
	Racial/ethnic minority population	−3.26 (−7.03 to 0.51)	0.080
	Income below 150% of the federal poverty level	9.30 (5.48 to 13.13)	<0.001
	No high school diploma	5.80 (0.90 to 10.69)	0.033
	Unemployment	8.95 (4.56 to 13.35)	0.002
Healthcare access and preventive care			
	Lack of health insurance	6.36 (−10.65 to 23.38)	0.265
	Routine checkup‡	10.35 (6.08 to 14.63)	0.003
	Up-to-date colorectal cancer screening	−9.61 (−14.20 to −5.02)	0.004
	Recent dental visit	−13.43 (−17.27 to −9.60)	<0.001
Behavioral risk indicators			
	Obesity	10.94 (8.29 to 13.60)	<0.001
	Current smoking	19.95 (18.08 to 21.82)	<0.001
	Binge drinking	−0.42 (−5.92 to 5.08)	0.848
	Short sleep duration	12.47 (10.35 to 14.60)	<0.001
	Physical inactivity	12.34 (7.88 to 16.79)	<0.001
Rurality			
	Nonmetropolitan county versus metropolitan county	13.93 (11.32 to 16.53)	<0.001

CI, confidence interval.

Each row was estimated in a separate population-weighted core-adjusted linear regression model. Models adjusted for the county proportion aged ≥65 years, nonmetropolitan county status, and state fixed effects. The rurality model adjusted for age and state fixed effects but did not self-adjust for rurality. Standard errors were clustered at the state level using the CR2 variance estimator, and inference was based on Satterthwaite degrees of freedom.

† Continuous county-level correlates were standardized to 1-SD increments. β coefficients represent absolute differences in age-adjusted county-level all-cancer mortality rates per 100,000 population associated with a 1-SD higher prevalence of the corresponding correlate. For rurality, the estimate compares nonmetropolitan with metropolitan counties. ‡ Routine checkup showed high focal-term variance inflation factors across outcomes and should be interpreted cautiously; see **Supplementary Table 3**.

Preventive care indicators showed inverse associations with all-cancer mortality. Higher CRC screening uptake was associated with lower all-cancer mortality (β = −9.61, 95% CI −14.20 to −5.02; P = 0.004), and higher recent dental visit prevalence was associated with lower all-cancer mortality (β = −13.43, 95% CI −17.27 to −9.60; P <0.001). Higher poverty, unemployment, single-parent household prevalence, obesity, short sleep duration, and physical inactivity were also associated with higher all-cancer mortality. Nonmetropolitan counties had higher all-cancer mortality rates than metropolitan counties (β = 13.93, 95% CI 11.32 to 16.53; P <0.001).

The overlap between CRC mortality and all-cancer mortality findings indicates that several contextual indicators, including broadband deprivation, lower preventive care engagement, smoking prevalence, and rurality, may reflect broader geographic cancer disadvantage. The observed associations should not be interpreted as evidence that these factors have disease-specific or causal effects.

### 3.4 Exploratory, sensitivity, collinearity, and spatial analyses

Core-adjusted associations with CRC incidence are presented in **Supplementary Table 1**. Several findings were directionally consistent with the CRC mortality analysis. Higher lack of broadband internet access was associated with higher CRC incidence (β = 2.76, 95% CI 2.33 to 3.20; P <0.001), whereas higher CRC screening uptake and recent dental visit prevalence were associated with lower CRC incidence. Higher current smoking prevalence, obesity, short sleep duration, physical inactivity, and nonmetropolitan county status were also associated with higher CRC incidence.

Sensitivity analyses using log(rate + 0.1) as the outcome are presented in **Supplementary Table 2**. The direction of associations for the principal exposures was generally consistent with the primary rate-scale analyses across CRC mortality, all-cancer mortality, and CRC incidence. For example, higher lack of broadband internet access and current smoking prevalence remained associated with higher rates, whereas higher CRC screening uptake and recent dental visit prevalence remained associated with lower rates. **Supplementary Figure 1** visually compares five principal core-adjusted associations across the three outcomes.

Variance inflation factor diagnostics are presented in **Supplementary Table 3**. Most focal exposure terms showed no material VIF concern. Up-to-date CRC screening showed moderate VIF values across outcomes, whereas routine checkup showed high VIF values. These findings support cautious interpretation of correlated healthcare-access indicators and reinforce the use of core-adjusted individual-exposure models as the primary framework for variable-level inference.

Residual spatial autocorrelation was detected for the primary core-adjusted models of the prespecified principal exposures. However, matched spatial error models produced directionally consistent estimates for lack of broadband internet access, CRC screening, recent dental visits, current smoking, and nonmetropolitan county status across CRC mortality, all-cancer mortality, and CRC incidence (**Supplementary Table 4**). **Supplementary Figure 2** compares the state-clustered CR2 and matched spatial error model estimates for CRC mortality.

Supplementary sequential conditional-association models for CRC mortality are presented in **Supplementary Table 5**. Estimates for several social and preventive care indicators changed after additional correlated domain variables were included. Because these models represent increasingly conditional specifications, they are presented for transparency and are not the primary basis for variable-level inference.

## 4 Discussion

In this nationwide county-level ecological study, we found substantial geographic and contextual variation in CRC mortality across U.S. counties. Counties in the highest quartile of CRC mortality had a less favorable social, preventive care, behavioral, and rurality profile than counties in the lowest quartile. In the primary core-adjusted individual-exposure models, higher prevalence of broadband deprivation, poverty, lower educational attainment, unemployment, current smoking, physical inactivity, and nonmetropolitan residence was associated with higher CRC mortality (5, 26). In contrast, higher CRC screening uptake and recent dental visit prevalence were associated with lower CRC mortality. These findings should be interpreted as county-level associations rather than independent effects or causal estimates. Several principal associations were directionally similar across CRC mortality, all-cancer mortality, and CRC incidence. Higher current smoking prevalence, lower CRC screening uptake, lower recent dental visit prevalence, and nonmetropolitan county status were associated with less favorable outcomes across the three measures. Lack of broadband internet access was positively associated not only with CRC mortality and CRC incidence but also with all-cancer mortality. Accordingly, the revised results do not support an interpretation that broadband deprivation is uniquely related to CRC outcomes. Instead, broadband access may function as a broader contextual marker of healthcare connectivity, social disadvantage, care navigation capacity, and geographic cancer burden.

The association of broadband deprivation with adverse cancer outcomes is plausibly related to the growing role of digital infrastructure in healthcare access and care coordination (27–29). Broadband access may facilitate use of patient portals, telehealth services, appointment scheduling, access to health information, communication with healthcare teams, and completion of multistep screening and follow-up pathways (27–29). CRC prevention and early detection frequently require several linked processes, including screening invitation, test completion, diagnostic evaluation after an abnormal result, colonoscopy, treatment referral, and surveillance (30–34). However, the present ecological analysis cannot establish whether county-level broadband access directly influences any individual patient’s screening, diagnosis, treatment, or survival.

The inverse associations of CRC screening and recent dental visits with CRC mortality should also be interpreted at the county level. CRC screening uptake may reflect both use of evidence-based screening and the availability of prevention-oriented healthcare infrastructure. Screening can reduce CRC burden through earlier detection and removal of precancerous lesions, although county-level screening prevalence cannot identify the timing, modality, completion, or quality of screening received by individual residents (30, 35–37). Recent dental visits are not a CRC prevention intervention. Rather, they may serve as a proxy for routine healthcare engagement, preventive care orientation, outpatient service access, or community-level healthcare connectivity (38, 39). Therefore, the association between dental visits and lower cancer burden should not be interpreted as evidence of a direct protective effect of dental care on CRC.

Current smoking showed strong positive associations with CRC mortality, all-cancer mortality, and CRC incidence. This pattern is consistent with smoking serving both as a known CRC-related risk factor and as a marker of adverse behavioral and chronic disease environments at the county level (40, 41). Other behavioral indicators, including obesity, short sleep duration, and physical inactivity, were also associated with higher CRC mortality in the core-adjusted analyses (42, 43). These findings support the importance of considering behavioral environments alongside structural and preventive care conditions when describing geographic cancer inequities. Routine checkup prevalence was positively associated with CRC mortality but showed high VIF values across outcomes; therefore, this estimate should be interpreted cautiously.

The supplementary analyses strengthened the robustness of the principal findings. Log-rate sensitivity analyses yielded generally similar directions of association for the key social, preventive care, behavioral, and rurality indicators. Although residual spatial autocorrelation was detected for the prespecified principal exposure models, matched spatial error models produced directionally consistent estimates for broadband deprivation, CRC screening, recent dental visits, current smoking, and nonmetropolitan county status. These results indicate that the main patterns were not materially altered when using the alternative spatial error specification. Nevertheless, neither the CR2 models nor the spatial error models establish causality or eliminate the possibility of residual spatially patterned confounding.

The comparison of CRC mortality with all-cancer mortality and CRC incidence provides useful descriptive context. The partial geographic overlap between outcomes suggests that some county-level indicators reflect broader cancer disadvantage, whereas the incomplete overlap suggests that CRC mortality, all-cancer mortality, and CRC incidence should not be treated as interchangeable outcomes. The outcome windows also differed slightly, with CRC incidence measured during 2018–2022 and mortality outcomes measured during 2019–2023. Therefore, cross-outcome comparisons should be interpreted cautiously and should not be considered formal evidence of disease-specific pathways.

From a public health perspective, the results support geographically targeted strategies that extend beyond individual-level risk modification. High-burden counties may benefit from multicomponent approaches that increase CRC screening uptake, strengthen follow-up after abnormal screening results, improve preventive care engagement, reduce smoking prevalence, address barriers to healthcare navigation, and improve access to services in nonmetropolitan communities (44–47). Digital inclusion may be relevant within such strategies, particularly when it supports care communication, screening navigation, and continuity of care; however, digital interventions should be implemented alongside, rather than instead of, broader efforts to reduce structural barriers and improve local healthcare capacity (27, 46). For rural counties with high poverty and limited broadband access, multicomponent strategies could combine affordable broadband expansion and digital navigation support with mailed or mobile stool-based CRC screening outreach, patient navigation for timely diagnostic colonoscopy after abnormal screening results, and stronger referral linkages between dental, primary care, and preventive services.

The key strengths of this study include the use of a comprehensive, multi-source national county-level dataset and a rigorous statistical framework incorporating population weighting, state fixed effects, CR2 clustered standard errors, and extensive spatial and sensitivity analyses. Several limitations should be acknowledged. First, the ecological and cross-sectional design precludes causal inference and raises the possibility of ecological fallacy; county-level associations should not be interpreted as person-level effects. Second, CRC incidence and mortality were measured during slightly different multi-year periods. Third, CRC mortality had more unavailable or suppressed county-level rates than the other outcomes, reducing the primary analytic sample and potentially introducing selection bias. Fourth, several variables, including broadband access, dental visits, and routine checkups, are proxies for broader contextual constructs and cannot identify the specific mechanisms responsible for observed associations. Fifth, the primary individual-exposure models were intentionally designed to avoid treating correlated social, preventive care, and behavioral variables as mutually independent predictors. Consequently, the estimated associations should not be interpreted as mutually adjusted effects across all domains. Finally, public county-level data did not permit assessment of tumor stage, treatment, person-level screening history, individual insurance status, or individual socioeconomic circumstances.

## 5 Conclusion

In this national county-level ecological analysis, higher CRC mortality was associated with adverse social and behavioral profiles, lower preventive care engagement, and nonmetropolitan residence. Higher prevalence of current smoking and greater broadband deprivation were associated with higher CRC mortality, whereas higher CRC screening uptake and recent dental visit prevalence were associated with lower CRC mortality in core-adjusted models. Similar directional patterns across CRC mortality, all-cancer mortality, and CRC incidence suggest that these contextual indicators may reflect broader geographic cancer disadvantage rather than CRC-specific mechanisms alone. Integrated structural, preventive care, behavioral, and rural health strategies may help reduce county-level disparities in CRC outcomes.

## Data Availability

The original contributions presented in the study are included in the article/**Supplementary Material**. Further inquiries can be directed to the corresponding author.
