# Supplementary material for: County-level social, preventive care, and behavioral correlates of colorectal cancer mortality in the United States: a nationwide ecological analysis
Source: Front Oncol. 2026 Jul 28;16:1844633. doi: 10.3389/fonc.2026.1844633 (PMC13457059; doi:10.3389/fonc.2026.1844633)
Supplement: Supplementary file 1 [file DataSheet1.docx]

**Supplementary to:**

**County-Level Social, Preventive Care, and Behavioral Correlates of Colorectal Cancer Mortality in the United States: A Nationwide Ecological Analysis**

**Kaide Xia^1†^, Li Luo^2†^, Chengdu Fan^3^, Hexuan Zhang^1^, Xiuju Yang^1^, Chaohuang Lin^4*^**

^1^ Laboratory of Molecular Genetic Diagnostics, Guiyang Maternal and Child Health Care Hospital, Guiyang Children's Hospital, Guiyang, China.

^2^ Department of Clinical Laboratory, The Second People's Hospital of Guiyang, Guiyang, China

^3^ Department of Anesthesiology, The Affiliated Cancer Hospital of Guizhou Medical University, Guiyang, China

^4^ Department of Gastrointestinal Surgery, The Affiliated Cancer Hospital of Guizhou Medical University, Guiyang, China

***Correspondence:**

Chaohuang Lin, Department of Gastrointestinal Surgery, The Affiliated Cancer Hospital of Guizhou Medical University, Guiyang, China. Email: [m15285933663_1@163.com](mailto:m15285933663_1@163.com)

^†^ K.X. and L.L. contributed equally to this work.

**Table S1. Core-adjusted associations of county-level correlates with colorectal cancer incidence, United States, 2018–2022 (n = 2,628 counties across 48 state clusters)**

| **Domain** | **County-level correlate†** | **β (95% CI), per 100,000** | **P value** |
| --- | --- | --- | --- |
| **Social determinants** |  |  |  |
|  | Crowded housing | 0.31 (−1.18 to 1.80) | 0.456 |
|  | Single-parent households | 1.41 (0.61 to 2.22) | 0.006 |
|  | Lack of broadband internet access | 2.76 (2.33 to 3.20) | <0.001 |
|  | High housing cost burden | 0.23 (−0.40 to 0.86) | 0.357 |
|  | Racial/ethnic minority population | 0.15 (−0.65 to 0.95) | 0.672 |
|  | Income below 150% of the federal poverty level | 1.59 (0.91 to 2.26) | 0.001 |
|  | No high school diploma | 1.57 (0.61 to 2.53) | 0.015 |
|  | Unemployment | 1.66 (0.90 to 2.42) | 0.001 |
| **Healthcare access and preventive care** |  |  |  |
|  | Lack of health insurance | 1.85 (−0.01 to 3.72) | 0.051 |
|  | Routine checkup‡ | 1.30 (−0.11 to 2.71) | 0.062 |
|  | Up-to-date colorectal cancer screening | −2.72 (−3.45 to −2.00) | <0.001 |
|  | Recent dental visit | −2.66 (−3.59 to −1.72) | <0.001 |
| **Behavioral risk indicators** |  |  |  |
|  | Obesity | 1.67 (1.01 to 2.33) | 0.004 |
|  | Current smoking | 3.23 (2.63 to 3.83) | <0.001 |
|  | Binge drinking | −0.83 (−1.62 to −0.03) | 0.045 |
|  | Short sleep duration | 2.20 (1.78 to 2.62) | <0.001 |
|  | Physical inactivity | 2.53 (1.90 to 3.16) | <0.001 |
| **Rurality** |  |  |  |
|  | Nonmetropolitan county versus metropolitan county | 3.98 (3.35 to 4.61) | <0.001 |

**Abbreviations:** CI, confidence interval; CRC, colorectal cancer. Each row was estimated in a separate population-weighted core-adjusted linear regression model. Models adjusted for the county proportion aged ≥65 years, nonmetropolitan county status, and state fixed effects. The rurality model adjusted for age and state fixed effects but did not self-adjust for rurality. Standard errors were clustered at the state level using the CR2 variance estimator, and inference was based on Satterthwaite degrees of freedom.

† Continuous county-level correlates were standardized to 1-SD increments. β coefficients represent absolute differences in age-adjusted county-level CRC incidence rates per 100,000 population associated with a 1-SD higher prevalence of the corresponding correlate. For rurality, the estimate compares nonmetropolitan with metropolitan counties. ‡ Routine checkup showed high focal-term variance inflation factors across outcomes and should be interpreted cautiously; see Table S3.

**Table S2. Log-rate sensitivity analyses using core-adjusted individual-exposure models**

**Panel A. CRC mortality, 2019–2023 (n = 2,126 counties across 49 state clusters)**

| **Domain** | **Variable** | **Percent change in rate (95% CI)** | **P value** | **Satterthwaite df** |
| --- | --- | --- | --- | --- |
| Social determinants | Crowded housing (%) | 0.92% (−4.98%, 7.18%) | 0.563 | 1.88 |
|  | Single-parent households (%) | 9.29% (4.16%, 14.67%) | 0.005 | 5.07 |
|  | Lack of broadband internet access (%) | 14.01% (10.66%, 17.47%) | <0.001 | 6.78 |
|  | High housing cost burden (%) | 4.28% (0.60%, 8.10%) | 0.032 | 3.71 |
|  | Racial/ethnic minority population (%) | 2.08% (−1.60%, 5.90%) | 0.228 | 7.12 |
|  | Income below 150% of poverty (%) | 9.93% (6.66%, 13.29%) | <0.001 | 6.12 |
|  | No high school diploma (%) | 8.04% (4.58%, 11.62%) | 0.005 | 2.86 |
|  | Unemployment (%) | 10.56% (6.04%, 15.28%) | <0.001 | 6.96 |
| Healthcare access / preventive care | Lack of health insurance (%) | 10.16% (−3.42%, 25.64%) | 0.090 | 2.17 |
|  | Routine checkup (%) | 7.87% (3.69%, 12.23%) | 0.006 | 3.98 |
|  | Up-to-date colorectal cancer screening (%) | −11.41% (−14.01%, −8.72%) | <0.001 | 4.24 |
|  | Recent dental visit (%) | −12.84% (−15.61%, −9.98%) | <0.001 | 5.14 |
| Behavioral risk indicators | Obesity (%) | 9.66% (7.90%, 11.44%) | <0.001 | 3.25 |
|  | Current smoking (%) | 16.99% (13.98%, 20.07%) | <0.001 | 8.50 |
|  | Binge drinking (%) | −3.31% (−7.13%, 0.66%) | 0.082 | 4.43 |
|  | Short sleep duration (%) | 12.13% (9.33%, 14.99%) | <0.001 | 7.42 |
|  | Physical inactivity (%) | 13.19% (9.54%, 16.96%) | <0.001 | 5.39 |
| Rurality | Nonmetropolitan county (vs metropolitan) | 19.14% (15.97%, 22.39%) | <0.001 | 21.44 |

**Panel B. All-cancer mortality, 2019–2023 (n = 3,005 counties across 49 state clusters)**

| **Domain** | **Variable** | **Percent change in rate (95% CI)** | **P value** | **Satterthwaite df** |
| --- | --- | --- | --- | --- |
| Social determinants | Crowded housing (%) | −2.96% (−7.53%, 1.85%) | 0.114 | 1.94 |
|  | Single-parent households (%) | 6.47% (2.99%, 10.08%) | 0.005 | 5.11 |
|  | Lack of broadband internet access (%) | 8.48% (5.38%, 11.68%) | <0.001 | 6.81 |
|  | High housing cost burden (%) | 2.15% (−1.04%, 5.44%) | 0.134 | 3.73 |
|  | Racial/ethnic minority population (%) | −2.50% (−5.33%, 0.43%) | 0.083 | 7.12 |
|  | Income below 150% of poverty (%) | 6.40% (3.57%, 9.31%) | 0.001 | 6.14 |
|  | No high school diploma (%) | 3.80% (0.26%, 7.47%) | 0.042 | 2.88 |
|  | Unemployment (%) | 6.04% (2.51%, 9.69%) | 0.005 | 7.04 |
| Healthcare access / preventive care | Lack of health insurance (%) | 4.44% (−6.92%, 17.20%) | 0.263 | 2.19 |
|  | Routine checkup (%) | 7.83% (4.65%, 11.11%) | 0.002 | 3.99 |
|  | Up-to-date colorectal cancer screening (%) | −6.22% (−9.20%, −3.15%) | 0.005 | 4.26 |
|  | Recent dental visit (%) | −8.61% (−11.05%, −6.10%) | <0.001 | 5.16 |
| Behavioral risk indicators | Obesity (%) | 8.19% (6.28%, 10.13%) | <0.001 | 3.26 |
|  | Current smoking (%) | 14.70% (12.94%, 16.50%) | <0.001 | 8.59 |
|  | Binge drinking (%) | 0.31% (−3.47%, 4.24%) | 0.838 | 4.44 |
|  | Short sleep duration (%) | 9.03% (7.15%, 10.95%) | <0.001 | 7.43 |
|  | Physical inactivity (%) | 8.69% (5.37%, 12.11%) | <0.001 | 5.40 |
| Rurality | Nonmetropolitan county (vs metropolitan) | 8.70% (6.78%, 10.66%) | <0.001 | 21.25 |

**Panel C. CRC incidence, 2018–2022 (n = 2,628 counties across 48 state clusters)**

| **Domain** | **Variable** | **Percent change in rate (95% CI)** | **P value** | **Satterthwaite df** |
| --- | --- | --- | --- | --- |
| Social determinants | Crowded housing (%) | 0.65% (−3.03%, 4.48%) | 0.519 | 1.92 |
|  | Single-parent households (%) | 3.92% (1.72%, 6.16%) | 0.006 | 5.07 |
|  | Lack of broadband internet access (%) | 7.22% (6.10%, 8.35%) | <0.001 | 6.73 |
|  | High housing cost burden (%) | 0.84% (−0.78%, 2.49%) | 0.218 | 3.71 |
|  | Racial/ethnic minority population (%) | 0.46% (−1.55%, 2.51%) | 0.609 | 7.09 |
|  | Income below 150% of poverty (%) | 4.13% (2.35%, 5.95%) | 0.001 | 6.06 |
|  | No high school diploma (%) | 4.19% (1.70%, 6.73%) | 0.013 | 2.85 |
|  | Unemployment (%) | 4.27% (2.31%, 6.27%) | 0.001 | 6.96 |
| Healthcare access / preventive care | Lack of health insurance (%) | 5.05% (−0.17%, 10.54%) | 0.054 | 2.15 |
|  | Routine checkup (%) | 3.76% (−0.21%, 7.89%) | 0.058 | 3.95 |
|  | Up-to-date colorectal cancer screening (%) | −6.82% (−8.50%, −5.12%) | <0.001 | 4.17 |
|  | Recent dental visit (%) | −6.68% (−8.76%, −4.56%) | <0.001 | 5.06 |
| Behavioral risk indicators | Obesity (%) | 4.62% (2.94%, 6.32%) | 0.003 | 3.22 |
|  | Current smoking (%) | 8.75% (7.22%, 10.30%) | <0.001 | 8.29 |
|  | Binge drinking (%) | −2.14% (−4.29%, 0.06%) | 0.054 | 4.40 |
|  | Short sleep duration (%) | 6.13% (5.03%, 7.23%) | <0.001 | 7.25 |
|  | Physical inactivity (%) | 6.87% (5.25%, 8.52%) | <0.001 | 5.29 |
| Rurality | Nonmetropolitan county (vs metropolitan) | 9.30% (7.58%, 11.04%) | <0.001 | 21.05 |

**Note:** Each row uses the same core-adjusted individual-exposure model as the main analysis, with log(rate + 0.1) as the outcome. Values are percentage changes in county-level rates associated with a 1-SD higher continuous exposure or nonmetropolitan versus metropolitan county status. Models were population-weighted and adjusted for the county proportion aged ≥65 years, nonmetropolitan county status, and state fixed effects; the nonmetropolitan-county model adjusted for age and state fixed effects but did not self-adjust for rurality. Standard errors were calculated using state-clustered CR2 estimation with Satterthwaite degrees of freedom.

**Table S3. Variable-specific VIFs for core-adjusted individual-exposure models**

| **Domain** | **Exposure** | **CRC mortality VIF** | **All-cancer mortality VIF** | **CRC incidence VIF** | **Interpretation*** |
| --- | --- | --- | --- | --- | --- |
| **Social determinants** | Crowded housing (%) | 3.17 | 3.04 | 3.06 | No material concern |
|  | Single-parent households (%) | 1.45 | 1.45 | 1.45 | No material concern |
|  | Lack of broadband internet access (%) | 1.78 | 1.82 | 1.82 | No material concern |
|  | High housing cost burden (%) | 1.50 | 1.53 | 1.52 | No material concern |
|  | Racial/ethnic minority population (%) | 2.93 | 2.91 | 2.92 | No material concern |
|  | Income below 150% of poverty (%) | 1.42 | 1.43 | 1.43 | No material concern |
|  | No high school diploma (%) | 1.68 | 1.67 | 1.67 | No material concern |
|  | Unemployment (%) | 1.41 | 1.40 | 1.40 | No material concern |
| **Healthcare access / preventive care** | Lack of health insurance (%) | 3.28 | 3.28 | 3.30 | No material concern |
|  | Routine checkup (%) | 10.84 | 10.80 | 10.92 | High VIF |
|  | Up-to-date colorectal cancer screening (%) | 6.40 | 6.27 | 6.39 | Moderate VIF |
|  | Recent dental visit (%) | 2.32 | 2.34 | 2.35 | No material concern |
| **Behavioral risk indicators** | Obesity (%) | 1.97 | 2.00 | 2.00 | No material concern |
|  | Current smoking (%) | 2.73 | 2.74 | 2.78 | No material concern |
|  | Binge drinking (%) | 3.04 | 3.10 | 3.10 | No material concern |
|  | Short sleep duration (%) | 2.08 | 2.10 | 2.11 | No material concern |
|  | Physical inactivity (%) | 2.08 | 2.11 | 2.12 | No material concern |
| **Rurality** | Nonmetropolitan county (vs metropolitan) | 1.37 | 1.42 | 1.41 | No material concern |

Note: Values are variance inflation factors for the focal exposure term in each core-adjusted individual-exposure model. Each continuous exposure was evaluated in a separate population-weighted model adjusted for the county proportion aged ≥65 years, nonmetropolitan county status, and state fixed effects. The nonmetropolitan-county model adjusted for age and state fixed effects but did not self-adjust for rurality. State-fixed-effect dummy terms are not displayed. VIF values of 5 to <10 were considered moderate, and values ≥10 were considered high. Routine checkup showed high VIFs, and up-to-date colorectal cancer screening showed moderate VIFs across outcomes; these findings were considered when selecting core-adjusted individual-exposure models as the primary framework for variable-level inference.

**Table S4. Key core-adjusted estimates: state-clustered CR2 versus matched spatial error models**

**Panel A. CRC mortality, 2019–2023 (n = 2,126 counties across 49 state clusters)**

| **Principal exposure** | **CR2 β (95% CI)** | **Spatial error model β (95% CI)** | **Residual Moran’s I (P value)** | **Spatial error λ** |
| --- | --- | --- | --- | --- |
| Lack of broadband internet access (%) | 1.82 (1.44, 2.20) | 1.87 (1.71, 2.04) | 0.129 (<0.001) | 0.251 |
| Up-to-date colorectal cancer screening (%) | −1.64 (−1.97, −1.31) | −1.60 (−1.78, −1.43) | 0.134 (<0.001) | 0.238 |
| Recent dental visit (%) | −1.84 (−2.27, −1.40) | −1.78 (−1.92, −1.65) | 0.107 (<0.001) | 0.198 |
| Current smoking (%) | 2.08 (1.74, 2.42) | 2.07 (1.93, 2.20) | 0.122 (<0.001) | 0.187 |
| Nonmetropolitan county (vs metropolitan) | 2.73 (2.35, 3.11) | 2.41 (2.09, 2.72) | 0.145 (<0.001) | 0.264 |

**Panel B. All-cancer mortality, 2019–2023 (n = 3,005 counties across 49 state clusters)**

| **Principal exposure** | **CR2 β (95% CI)** | **Spatial error model β (95% CI)** | **Residual Moran’s I (P value)** | **Spatial error λ** |
| --- | --- | --- | --- | --- |
| Lack of broadband internet access (%) | 12.28 (7.98, 16.57) | 13.34 (12.37, 14.32) | 0.168 (<0.001) | 0.500 |
| Up-to-date colorectal cancer screening (%) | −9.61 (−14.20, −5.02) | −10.41 (−11.47, −9.35) | 0.193 (<0.001) | 0.518 |
| Recent dental visit (%) | −13.43 (−17.27, −9.60) | −13.03 (−13.83, −12.22) | 0.149 (<0.001) | 0.482 |
| Current smoking (%) | 19.95 (18.08, 21.82) | 19.35 (18.68, 20.02) | 0.088 (<0.001) | 0.276 |
| Nonmetropolitan county (vs metropolitan) | 13.93 (11.32, 16.53) | 11.25 (9.33, 13.16) | 0.191 (<0.001) | 0.495 |

**Panel C. CRC incidence, 2018–2022 (n = 2,628 counties across 48 state clusters)**

| **Principal exposure** | **CR2 β (95% CI)** | **Spatial error model β (95% CI)** | **Residual Moran’s I (P value)** | **Spatial error λ** |
| --- | --- | --- | --- | --- |
| Lack of broadband internet access (%) | 2.76 (2.33, 3.20) | 2.81 (2.52, 3.10) | 0.161 (<0.001) | 0.260 |
| Up-to-date colorectal cancer screening (%) | −2.72 (−3.45, −2.00) | −2.60 (−2.92, −2.29) | 0.165 (<0.001) | 0.244 |
| Recent dental visit (%) | −2.66 (−3.59, −1.72) | −2.58 (−2.83, −2.32) | 0.161 (<0.001) | 0.249 |
| Current smoking (%) | 3.23 (2.63, 3.83) | 3.22 (2.97, 3.47) | 0.153 (<0.001) | 0.239 |
| Nonmetropolitan county (vs metropolitan) | 3.98 (3.35, 4.61) | 3.61 (3.06, 4.16) | 0.174 (<0.001) | 0.274 |

**Note:** CR2 estimates were obtained from the primary population-weighted core-adjusted individual-exposure models with state fixed effects and state-clustered CR2 standard errors. Matched spatial error models used the identical outcome, exposure, covariates, state fixed effects, population weights, and queen-contiguity county neighbor structure. Residual Moran’s I was calculated from the corresponding CR2 model before fitting the spatial error model. Continuous exposures were standardized to 1-SD increments. β coefficients represent absolute differences in county-level rates per 100,000 population. Across all principal exposures and outcomes, CR2 and spatial error model estimates were directionally consistent.

**Table S5. Sequential conditional-association models for CRC mortality (n = 2,126 counties across 49 state clusters)**

| **Domain** | **Variable** | **Model 1, β (95% CI); P value** | **Model 2, β (95% CI); P value** | **Model 3, β (95% CI); P value** |
| --- | --- | --- | --- | --- |
| **Social determinants** | Crowded housing (%) | −0.69 (−1.43, 0.05); 0.057 | −0.62 (−1.25, 0.00); 0.051 | −0.30 (−0.72, 0.13); 0.104 |
|  | Single-parent households (%) | 0.38 (−0.11, 0.87); 0.099 | 0.34 (−0.08, 0.75); 0.085 | 0.04 (−0.36, 0.44); 0.800 |
|  | Lack of broadband internet access (%) | 1.09 (0.67, 1.52); <0.001 | 0.70 (0.33, 1.08); 0.001 | 0.45 (0.06, 0.83); 0.026 |
|  | High housing cost burden (%) | −0.09 (−0.50, 0.33); 0.538 | −0.09 (−0.53, 0.34); 0.525 | −0.06 (−0.48, 0.37); 0.727 |
|  | Racial/ethnic minority population (%) | −0.44 (−0.76, −0.12); 0.017 | −0.36 (−0.69, −0.03); 0.039 | 0.13 (−0.39, 0.65); 0.576 |
|  | Income below 150% of poverty (%) | 0.28 (−0.14, 0.71); 0.154 | −0.16 (−0.65, 0.33); 0.449 | −0.42 (−1.14, 0.30); 0.204 |
|  | No high school diploma (%) | 0.61 (−0.03, 1.25); 0.057 | −0.14 (−0.52, 0.24); 0.401 | −0.42 (−1.14, 0.30); 0.191 |
|  | Unemployment (%) | 0.58 (0.25, 0.90); 0.004 | 0.32 (−0.02, 0.67); 0.061 | 0.17 (−0.14, 0.47); 0.244 |
| **Healthcare access / preventive care** | Lack of health insurance (%) | — | 0.04 (−0.79, 0.86); 0.894 | 0.05 (−1.74, 1.84); 0.937 |
|  | Routine checkup (%) | — | 0.48 (0.11, 0.85); 0.025 | 0.10 (−0.23, 0.44); 0.427 |
|  | Up-to-date colorectal cancer screening (%) | — | −0.65 (−0.93, −0.37); <0.001 | −0.55 (−0.86, −0.24); 0.004 |
|  | Recent dental visit (%) | — | −1.46 (−2.07, −0.84); 0.002 | −0.70 (−1.20, −0.20); 0.014 |
| **Behavioral risk indicators** | Obesity (%) | — | — | 0.13 (−0.34, 0.60); 0.428 |
|  | Current smoking (%) | — | — | 1.14 (0.04, 2.24); 0.045 |
|  | Binge drinking (%) | — | — | 0.14 (−0.13, 0.41); 0.269 |
|  | Short sleep duration (%) | — | — | 0.20 (−0.22, 0.62); 0.283 |
|  | Physical inactivity (%) | — | — | 0.25 (−0.26, 0.75); 0.272 |

**Note:** Model 1 included social determinants, the county proportion aged ≥65 years, nonmetropolitan county status, and state fixed effects. Model 2 additionally included healthcare access and preventive-care indicators. Model 3 additionally included behavioral risk indicators. All models were population-weighted and used state-clustered CR2 standard errors with Satterthwaite degrees of freedom. Continuous variables were standardized to 1-SD increments. β coefficients represent absolute differences in county-level CRC mortality rates per 100,000 population. These sequential conditional-association models are reported for transparency only. Because social, preventive-care, and behavioral indicators were correlated at the county level, particularly in the fully adjusted specification, Model 3 is not the primary basis for variable-level inference. No formal mediation analysis was undertaken, and changes in estimates across the sequential models were not interpreted as evidence of mediation, confounding control, or independent explanatory power.

**Figure S1. Key core-adjusted associations across cancer outcomes**

Estimates are from separate population-weighted core-adjusted individual-exposure models for CRC mortality (2019–2023), all-cancer mortality (2019–2023), and CRC incidence (2018–2022). Continuous exposures are expressed per 1-SD higher county-level prevalence; nonmetropolitan county status is compared with metropolitan county status. Models adjusted for the county proportion aged ≥65 years, nonmetropolitan county status, and state fixed effects; the rurality model adjusted for age and state fixed effects but did not self-adjust for rurality. Standard errors were clustered at the state level using CR2 estimation with Satterthwaite degrees of freedom. Points indicate β coefficients and horizontal bars indicate 95% confidence intervals. β coefficients represent absolute differences in county-level rates per 100,000 population. The x-axis scale differs across outcome panels.

**Figure S2. CRC mortality: state-clustered CR2 versus spatial error model**

Estimates compare the primary state-clustered CR2 models with spatial error models for CRC mortality during 2019–2023. Each pair used an identical population-weighted core-adjusted individual-exposure specification, including the focal exposure, the county proportion aged ≥65 years, nonmetropolitan county status, and state fixed effects; the rurality model adjusted for age and state fixed effects but did not self-adjust for rurality. Continuous exposures are expressed per 1-SD higher county-level prevalence, whereas nonmetropolitan county status is compared with metropolitan county status. Spatial error models used a queen-contiguity county neighbor structure. Points indicate β coefficients and horizontal bars indicate 95% confidence intervals. β coefficients represent absolute differences in county-level CRC mortality rates per 100,000 population.
